# Hybrid Si nanocones/PEDOT:PSS solar cell

**DOI:** 10.1186/s11671-015-0891-6

**Published:** 2015-04-21

**Authors:** Hao Wang, Jianxiong Wang, ᅟ Rusli

**Affiliations:** Novitas, Nanoelectronics Centre of Excellence, School of Electrical and Electronic Engineering, Nanyang Technological University, 50 Nanyang Avenue, Singapore, 639798 Singapore; CINTRA UMI CNRS/NTU/THALES 3288, Research Techno Plaza, 50 Nanyang Drive, Border X Block, Level 6, Singapore, 637553 Singapore

**Keywords:** Hybrid solar cell, Silicon nanocones, Conductive polymer, Optical simulation

## Abstract

Periodic silicon nanocones (SiNCs) with different periodicities are fabricated by dry etching of a Si substrate patterned using monolayer polystyrene (PS) nanospheres as a mask. Hybrid Si/PEDOT:PSS solar cells based on the SiNCs are then fabricated and characterized in terms of their optical, electrical, and photovoltaic properties. The optical properties of the SiNCs are also investigated using theoretical simulation based on the finite element method. The SiNCs reveal excellent light trapping ability as compared to a planar Si substrate. It is found that the power conversion efficiency (PCE) of the hybrid cells decreases with increasing periodicity of the SiNCs. The highest PCE of 7.1% is achieved for the SiNC hybrid cell with a 400-nm periodicity, due to the strong light trapping near the peak of the solar spectrum and better current collection efficiency.

**PACS:** 81.07.-b; 81.16.-c; 88.40.hj

## Background

Despite the significant progress achieved for silicon (Si) solar cell in the past several decades, its wide application is still impeded by its high cost. Since the cost of the Si material constitutes about 50% of the total cost of the solar cell, it is imperative that thin Si layer instead of bulk Si be adopted in solar cell to reduce the usage of Si material and lower the cost [1]. However, reducing the thickness of the Si layer requires improved light absorption to ensure that the amount of sunlight absorbed is not compromised. In recent years, this has been achieved by incorporating Si solar cells with nanostructures such as nanowires [2-4], nanoholes [5], and nanocones [1,6] to trap sunlight effectively and result in improved light absorption. Besides the issue of material cost, simplification of the fabrication processes of Si solar cell presents another possible way to address the cost concern. In this aspect, the hybrid Si/organic solar cells that have emerged in recent years provide a potential low-cost alternative to the conventional Si solar cell [7-10]. Unlike the conventional Si solar cell that requires an expensive and high-temperature process to form the p-n junction, the junctions in the hybrid cells are formed by a low-temperature solution-based process that leverages on the advantages of organic materials, which thus greatly simplifies the fabrication process. The convergence of the above two ideas on cost reduction suggests hybrid Si/organic solar cells based on thin-film Si incorporated with nanostructure as a promising approach to lower the cost of solar cells without compromising on their efficiency [3].

Currently, Si/poly(3,4-ethylenedioxythiophene):poly(styrenesulfonate) (PEDOT:PSS) is the type of hybrid cell that is most commonly investigated. In the past few years, Si/PEDOT:PSS hybrid cells incorporated with Si nanostructures, in particular Si nanowires (SiNWs), have been extensively studied, and their power conversion efficiencies (PCE) have greatly improved to 13% [11,12]. To date, most of the hybrid SiNW solar cells are typically based on SiNWs fabricated using the metal-catalyzed electroless etching (MCEE) technique [4,13]. Such SiNWs are not periodic, and thus it is difficult to study and understand the detailed effects of the nanostructural parameters on the performance of the cells. Besides, there is limited control on the dimensions of the SiNWs, such as diameter and periodicity, to allow optimization of the cell performance. In addition, longer SiNWs fabricated from the solution-based MCEE technique suffers from agglomeration due to the effect of surface tension [14]. To better understand the effects of the structural parameters on the cell performance and to optimize the cell efficiency, it is imperative that hybrid solar cells based on periodic nanostructures with controllable dimensions be studied. Many top-down approaches have been developed to fabricate periodic nanostructures, which include deep-UV lithography [15], electronic beam lithography, focused ion beam (FIB) writing, and nanoimprinting [16,17]. Bottom-up approaches such as the vapor-liquid-solid (VLS) growth of periodic nanostructures have also been extensively studied [18]. However, these techniques either require the use of expensive equipment or involve complicated and time-consuming processes. Moreover, methods such as the e-beam lithography, FIB, and VLS growth also suffer from low throughput. Compared to these methods, nanosphere lithography (NSL) is a promising low-cost approach that is capable of fabricating periodic and homogenous nanostructures of various sizes at the wafer scale [19].

In this study, we demonstrate hybrid Si/PEDOT:PSS solar cells based on periodic Si nanocones (SiNCs) with different periodicities that range from 400 to 800 nm. The SiNCs are fabricated by dry etching of a Si substrate using assembled monolayer polystyrene (PS) nanospheres as a mask. Compared with SiNWs, SiNCs are mechanically more robust due to the larger base. Besides, their structure presents a more gradual change in the effective refractive index and thus is expected to possess better antireflective property [20]. The hybrid SiNC/PEDOT:PSS solar cell also exhibits improved optical properties and short-circuit current density (*J*_sc_) as compared to planar Si hybrid cells [21]. Besides experimental investigation of the SiNC hybrid solar cells, their optical properties are also studied theoretically using a simulation based on the finite element method. We found that the SiNC/PEDOT:PSS solar cell with a periodicity of 400 nm presents the highest PCE of 7.1% due to its strong light trapping around the peak of the solar spectrum and better current collection efficiency.

## Methods

**Figure 1:**
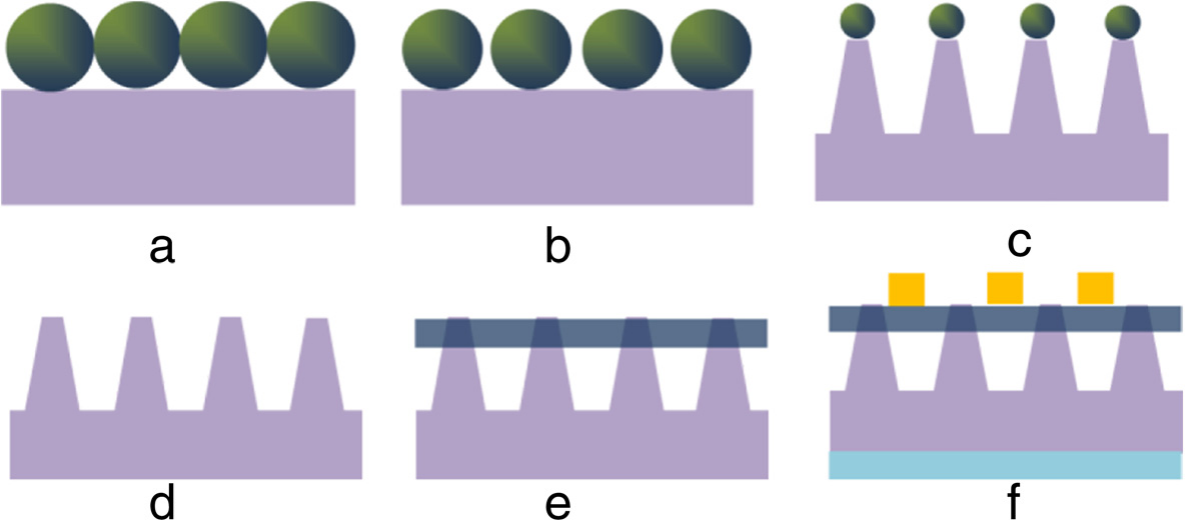
Schematic diagrams illustrating the fabrication process for SiNC/PEDOT:PSS hybrid cell. **(a)** Monolayer PS nanospheres prepared on an n-type Si substrate. **(b)** O_2_ plasma treatment of the PS nanospheres to reduce their diameter. **(c)** Cl_2_ plasma etching of the Si substrate to form SiNCs. **(d)** Removal of the PS nanospheres. **(e)** Spin coating of the PEDOT:PSS layer on the SiNCs. **(f)** Deposition of silver grid and Ti/Pd/Ag as the front and rear electrodes, respectively, to complete the hybrid cell.

The SiNCs were fabricated by the nanosphere lithography technique as illustrated in Figure 1. PS nanospheres were first deposited as a monolayer on an n-type (100) Si substrate as shown in Figure 1a. Briefly, the PS nanosphere solution was first mixed with ethanol in a volume ratio of 1:1 and then dropped on the water surface in a Petri dish. The nanospheres assembled into a monolayer and floated on the water and were subsequently transferred to the Si substrate by the Langmuir-Blodgett assembly method. To obtain SiNC arrays with different periodicities, PS nanospheres with different diameters of 400, 500, 600, and 800 nm were used. Note that the diameter determines the periodicity of the nanocones formed ultimately. The PS nanospheres were first etched using O_2_ plasma to reduce their diameters (Figure 1b) under the conditions of 30-W RF power, 20-sccm O_2_ gas flow rate, and 200-mTorr pressure. Following that, chlorine (Cl_2_) plasma was used to etch the Si substrate masked by the PS nanospheres to form SiNC arrays (Figure 1c). The Cl_2_ plasma etching was conducted for 5 min with a Cl_2_ flow rate of 50 sccm under a RF power of 200 W and pressure of 160 mTorr. Note that the nanospheres were also etched in the process of the Si etching. After the etching process, the nanospheres were removed by the organic solvent toluene (Figure 1d). For the fabrication of the hybrid cells, PEDOT:PSS (PH1000 from Clevios) solution mixed with 5 wt % dimethyl sulfoxide and 1 wt % Triton 100 was deposited on the SiNCs by spin coating at 3,000 rpm for 50 s (Figure 1e). The thickness of the PEDOT:PSS film coated is about 50 nm. Silver grid electrode was then deposited on top of the PEDOT:PSS layer and Ti/Pd/Ag electrode (50/50/1,000 nm) on the rear surface of the Si substrate to complete the hybrid solar cell structure (Figure 1f).

## Results and discussion

**Figure 2:**
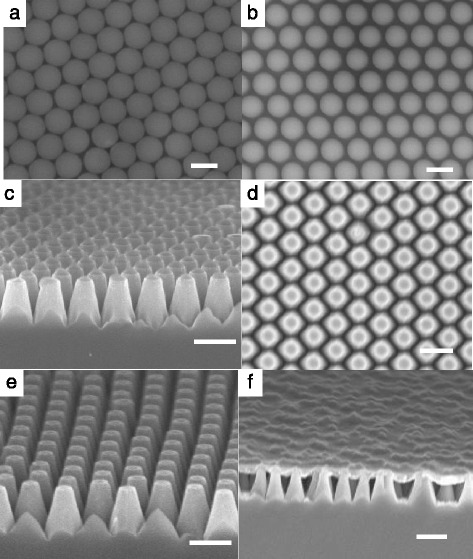
SEM images of the SiNC/PEDOT:PSS solar cell at various stages of the fabrication process. **(a)** Monolayer PS nanospheres prepared on a Si substrate. **(b)** PS nanospheres after the O_2_ etching process. **(c)** Formation of periodic nanocones after the Cl_2_ plasma etching process. **(d)** Top and **(e)** side views of the SiNCs after the removal of the nanospheres. **(f)** PEDOT:PSS layer spin-coated on the nanocones. The scale bar is 400 nm.

Figure 2 shows the scanning electron microscope (SEM) images of the SiNC/PEDOT:PSS solar cell fabricated at various stages. Figure 2a presents a typical SEM image of the monolayer PS nanospheres deposited on a Si substrate. The nanospheres with a diameter of 400 nm are in contact with one another and form a hexagonal pattern. After the O_2_ plasma etching, the diameter of the PS nanospheres was reduced to about 330 nm, as seen in Figure 2b. It is typically just slightly less than the original diameter of the PS nanospheres, and it approximately defines the bottom diameter of SiNCs formed. Figure 2c displays the SiNCs formed after the Cl_2_ plasma etching process, while Figure 2d,e shows the top and side views, respectively, of the SiNCs after the removal of the nanospheres. Note that the diameter of PS nanospheres after the Cl_2_ plasma etching approximately defines the top diameter of SiNCs formed. From the images, it is noted that the SiNCs have a height of about 500 nm and the top diameter is about half of the bottom diameter. The periodicity, which is determined by the original size of the nanosphere, is 400 nm for this sample. The SiNCs fabricated with PS nanospheres of diameters of 500, 600, and 800 nm all have about the same height of about 500 nm as controlled by the Si etching time, and the ratio of the top to bottom diameters is about 0.5. Shown in Figure 2f is the image of the PEDOT:PSS layer spin coated on the SiNCs. Due to its large molecular chain, it forms a layer on top and does not penetrate into the SiNCs.

**Figure 3:**
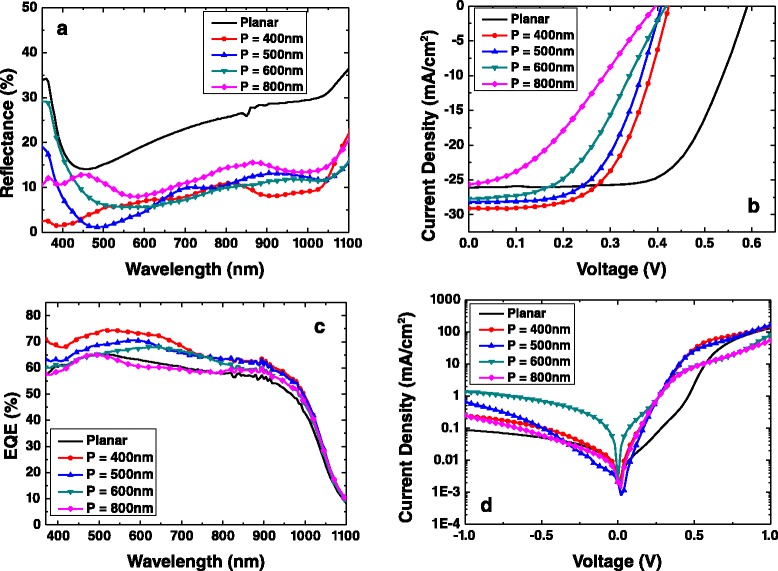
Reflectance spectra of planar and SiNC samples, current density-voltage characteristics, and external quantum efficiency. **(a)** Reflectance spectra of planar and SiNC samples coated with PEDOT:PSS, with periodicities of 400, 500, 600, and 800 nm. **(b)** Current density-voltage (*J*-*V*) characteristics, **(c)** external quantum efficiency (EQE), and **(d)** current density-voltage (*J*-*V*) characteristics under dark condition of the hybrid cells.

Figure 3a shows the reflectance spectra of the SiNC structures with periodicities (*P*) of 400, 500, 600, and 800 nm coated with PEDOT:PSS, together with that of a planar Si cell, recorded using an UV/Vis/NIR spectrophotometer system (Lambda 950). It is seen that the reflectance of all the SiNC samples is generally low, mostly in the range of 5% to 15% over a wide wavelength spectrum of 350 to 1,100 nm. It is also much lower than that of the planar cell, reaffirming the light trapping enhancement by the SiNCs. Figure 3b shows the current density-voltage (*J*-*V*) characteristics of the SiNC hybrid cells with different periodicities. The *J*-*V* curves were recorded under simulated AM 1.5 G irradiation at 100 mW/cm^2^. The photovoltaic parameters of short-circuit current density (*J*_sc_), open-circuit voltage (*V*_oc_), fill factor (FF), and PCE extracted from the *J*-*V* curves are summarized in Table 1. It is seen that the highest PCE of 7.1% is obtained for the cell with *P* = 400 nm. With an increase in the periodicity, the *V*_oc_ is relatively stable whereas both the *J*_sc_ and FF decrease, resulting in a drop in the PCE. The PCE of the hybrid cell with *P* = 800 nm is decreased to only 3.6%.

**Table 1:** **Photovoltaic parameters of the hybrid cells based on planar Si and SiNCs with different periodicities**

**Sample**	***J***_**sc**_ **(mA/cm** ^**2**^ **)**	***V***_**oc**_ **(V)**	**FF (%)**	**PCE (%)**
Planar	26.1	0.59	64.9	10.0
*P* 400 nm	29.1	0.42	57.7	7.1
*P* 500 nm	28.2	0.41	56.7	6.5
*P* 600 nm	27.7	0.42	45.8	5.3
*P* 800 nm	25.7	0.40	35.3	3.6

In terms of the photovoltaic parameters, the SiNC cells of smaller periodicities exhibit a better *J*_sc_ than the planar cell. For example, the one with *P* = 400 nm has a *J*_sc_ of 29.1 mA/cm^2^ in comparison to that of a planar cell of 26.1 mA/cm^2^. On the other hand, the *V*_oc_ of the SiNC hybrid cells is only about 0.42 V, which is much lower than the *V*_oc_ of 0.59 V observed for the planar cell [21]. This is attributed to the high recombination rate of the SiNC hybrid cells as compared to that of the planar cell. The Cl_2_ plasma etching and the ion bombardment will introduce surface defects at the SiNCs that act as recombination sites and promote carrier recombination. This and the larger surface area of the SiNCs are responsible for the increased carrier recombination. The degradation in *V*_oc_ observed for the SiNC hybrid cells is also corroborated by the increased reverse saturation current density (*J*_0_) of the dark *J*-*V* curve shown in Figure 3d, which is up to 1 order of magnitude higher than that of the planar cell. This issue can be addressed by proper surface treatment and passivation to minimize the defects [21-23]. Figure 3c shows the external quantum efficiency (EQE) of the hybrid solar cells where it is seen that the cell with *P* = 400 nm exhibits the strongest spectral response, particularly at a shorter wavelength range. Overall, the EQE decreases slightly with increasing periodicity, which is consistent with the *J*_sc_ trend as observed in Table 1.

**Figure 4:**
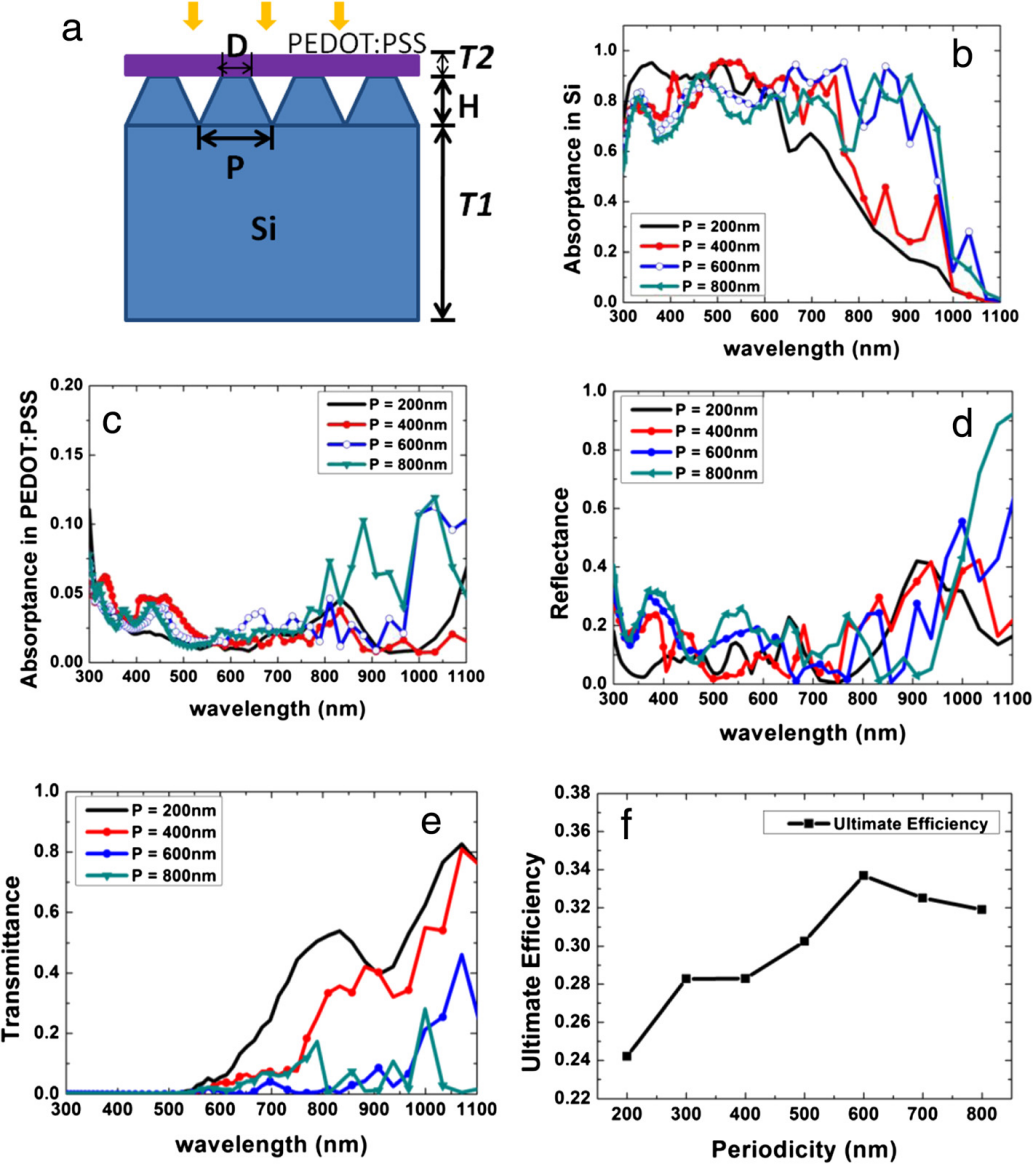
Optical simulation structure and results of the SiNC/PEDOT:PSS hybrid cell. **(a)** Schematic diagram of the simulated SiNC/PEDOT:PSS structure. Simulated light absorption in the **(b)** Si layer and **(c)** PEDOT:PSS layer. **(d)** Reflectance and **(e)** transmission spectra of the hybrid structure. **(f)** Ultimate efficiency of the hybrid structure as a function of *P* at a fixed *D*/*P* ratio of 0.5.

To further understand the optical properties of the hybrid SiNC/PEDOT:PSS cells, a simulation study of their optical characteristics has been performed using the commercial software High Frequency Structural Simulator based on the finite element method [24]. Figure 4a shows the schematic of the simulated hybrid SiNC/PEDOT:PSS structure. It consists of periodic SiNCs with a height (*H*) of 500 nm that is approximately the value observed experimentally for the SiNCs fabricated. There is an underlying Si thin film with a thickness (T1) of 3 μm and a top PEDOT:PSS layer with a thickness (T2) of 50 nm. The top diameter (*D*) to periodicity (*P*) ratio (*D*/*P* ratio) of the SiNCs is fixed at 0.5, similarly based on the experimental value, while *P* is varied from 200 to 800 nm to find the optimal structure that maximizes sunlight absorption. A plane wave with a wavelength *λ* ranging from 300 to 1,100 nm is incident normally on top of the hybrid structure. The periodic structure is simulated by applying periodic boundary conditions to the unit cell. The refractive indices of Si and PEDOT:PSS are obtained from the literature [25,26].

Figure 4b,c depicts the simulated light absorption in the Si and PEDOT:PSS materials, respectively, whereas Figure 4d,e shows the simulated overall reflectance and transmittance spectra of the hybrid structures, respectively. At the smallest *P* of 200 nm, the light absorption is enhanced at a shorter wavelength with *λ* < 550 nm, attributed to the strong scattering resulting from the comparable size of the structural dimension and the light wavelength [27]. As *P* is increased to 400 nm, the light absorption peak shifts to 550 nm and there is also a significant increase in the light absorption for long wavelength light (*λ* > 600 nm) compared to the *P* = 200 nm structure. This is due to the increase in the structural dimension which correspondingly shifts the strong scattering to longer wavelengths. When *P* is 600 nm, though there is a slight drop in the light absorption for a shorter wavelength range below 600 nm, the light absorption is significantly enhanced for *λ* > 600 nm as compared to the structures with smaller *P*. The strong scattering in the longer wavelength range increases the optical path length and results in a low transmittance, as can be seen in Figure 4e. This indicates that the light is strongly absorbed before it could penetrate through the structure. As *P* is further increased to 800 nm, the structure exhibits a slightly higher light absorption at a longer wavelength as compared to the *P* = 600 nm structure, but the absorption is generally lower for the *λ* < 800 nm range, resulting in a degradation of its overall performance. It is noted that as *P* increases, the light absorption in the PEDOT:PSS layer is strong for the structures with *P* = 600 and 800 nm, as there is a strong scattering at the longer wavelength range of *λ* > 800 nm where the absorption coefficients of PEDOT:PSS are higher.

Figure 4f summarizes the ultimate efficiencies for the simulated structures as a function of *P* at a fixed *D*/*P* ratio of 0.5. The ultimate efficiency is calculated from the absorption spectra under the standard air mass 1.5 spectrum [28] to determine the optimum structure for a maximum sunlight harvesting. It is given by [29]:

$$ \eta =\frac{{\displaystyle {\int}_{\mathrm{Eg}}^{\infty }}\frac{I(E)\times \alpha (E)\times \mathrm{Eg}}{E}dE}{{\displaystyle {\int}_0^{\infty }}I(E)dE} $$

where *I*(*E*) is the spectral solar intensity corresponding to the air mass 1.5 direct normal and circumsolar spectrum [28], *E* is the photon energy, Eg is the Si band gap energy of 1.1 eV, and *α*(*E*) is the absorption spectrum of the hybrid structure. This formula assumes that each photon with energy greater than the band gap will be absorbed in the solar cell to generate one electron-hole pair, which is then extracted as an electric current without loss [29]. From Figure 4f, it is seen that the SiNC/PEDOT:PSS hybrid structure reveals the highest ultimate efficiency of 33.7% at *P* = 600 nm, which is attributed to the enhancement in light absorption over a broad wavelength range that includes the peak of the solar spectrum.

It is noted that the simulated result is not exactly consistent with the experimental result, as the highest efficiency for the latter is found to occur for the structure with a smaller *P* of 400 nm. The deviation can be attributed to the fact that our simulation was performed for a Si thin film of a 3-μm thickness, whereas the actual devices were fabricated on thick Si substrates of 575 μm. Note that due to computing resource constraints, it is challenging to simulate structures with thick Si substrates. Due to the different Si absorbing layer thicknesses, the overall absorption characteristics and hence the optimum structural parameter will differ. Indeed as can be seen from Figure 4e, the structures with smaller *P* of 200 and 400 nm have high transmittance at a longer wavelength of *λ* > 600 nm, attributed to the lower absorption coefficients at these wavelengths and the thin Si absorbing layer. However, this is not the case when thick Si substrates are used as the light will be fully absorbed. Therefore, the maximum absorption is expected to occur at larger *P* for the simulated Si thin film structure. Note that for low-cost practical application, the hybrid cells should be fabricated using Si thin films instead of bulk Si wafers. Thus, our simulation results are relevant and reveal how the structures should be optimized when Si thin films are employed. Another factor that accounts for the variation between the experimental and simulated results is that the fabricated structures are not perfectly periodic as noted from Figure 2f, in contrast to the simulated structures.

**Figure 5:**
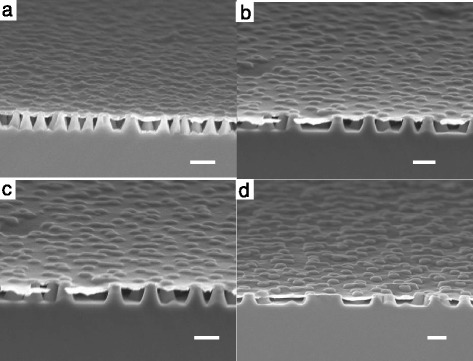
Surface morphology of the PEDOT:PSS layer on the top of the Si nanocones. With different periodicities of **(a)** 400 nm, **(b)** 500 nm, **(c)** 600 nm, and **(d)** 800 nm. The scale bar is 500 nm.

Our experimental results indicate that *J*_sc_ decreases with increasing periodicity, whereas the simulation results reveal that the ultimate efficiency increases with periodicity up to *P* = 600 nm. The inconsistent trend in the change in *J*_sc_ and optical absorption may be associated with the poorer current collection efficiency for the experimental cells at a larger periodicity. As the PEDOT:PSS layer sits on top of the SiNCs in contact with their sharp tips, some may pierce through the PEDOT:PSS layer and come in contact directly with the top silver grid to result in lower shunt resistance. This is more likely to occur for structures with larger *P* as there is more prominent protruding of the SiNCs on the PEDOT:PSS layer, as can be seen from Figure 5 that shows the surface morphology of the PEDOT:PSS layer on the top of the SiNCs with different *P*. Such structures also have rougher surfaces as the PEDOT:PSS layer is not well supported by the nanocone tips at larger *P* and sinks because of gravity. The rougher surface will render it harder to form continuous Ag grid on top and also result in poorer contact between the Ag grid and the PEDOT:PSS layer. This will lead to higher series resistance of the cells. Therefore, the irregular surface morphology of the structures with larger *P* will lead to degradation in *J*_sc_ and the FF as observed experimentally. This also explains why the optimum *P* for the experimental cells is observed at a lower value compared to that deduced from the optical simulation.

## Conclusions

In summary, periodic SiNCs have been fabricated by dry etching of the Si substrate using patterned PS nanospheres as a mask. Hybrid SiNC/PEDOT:PSS solar cells with different SiNC periodicities have been fabricated and characterized. Excellent light trapping ability of the SiNCs has been demonstrated by both the experimental results and theoretical simulation. The highest PCE of 7.1% has been achieved for the hybrid cell with a periodicity of 400 nm, due to the strong light trapping near the peak of the solar spectrum and good current collected efficiency. With proper surface treatment and passivation to minimize the defects on the SiNC surface, it is expected that the performance of the cells can be further improved. The fabrication process employed in this work can also be readily applied to a Si thin film to realize low-cost and highly efficient hybrid Si/PEDOT:PSS thin film cells based on SiNCs.
